# Sponge Counting

**Published:** 1900-05-05

**Authors:** 


### SPONGE COUNTING.

Dr. Wengebauer, of Warsaw, has recently done a good piece of work in collecting a long series of cases in which, in closing the wound after laparotomy, sponges and instruments of one kind and another have been accidentally left in the abdomen. He has got together 101 cases, in 38 of which the foreign body was only found at the necropsy. Among the more curious of the bodies left in the abdomen was a signet ring. This was removed from Douglas's pouch by operation through the vagina two years after a laparotomy. That a surgeon should operate with rings on his fingers is a strange commentary on all that we hear about modern antiseptic precautions. It is rather interesting to note that the known liability of sponges, forceps, and other things to be lost in the abdomen leads sometimes to a sort of scare on the subject. The idea is somehow suggested that something has been left; the operator going over in his mind every step of the operation is unable to shake off the feeling that it is so; and he therefore reopens the wound but finds nothing. This occurred twice. The importance of a careful count of both instruments and sponges cannot be exaggerated.

